# *FGFR1 *amplification in breast carcinomas: a chromogenic *in situ *hybridisation analysis

**DOI:** 10.1186/bcr1665

**Published:** 2007-03-30

**Authors:** Somaia Elbauomy Elsheikh, Andrew R Green, Maryou BK Lambros, Nicholas C Turner, Matthew J Grainge, Des Powe, Ian O Ellis, Jorge S Reis-Filho

**Affiliations:** 1 Department of Histopathology, School of Molecular Medical Sciences, A Floor, West Block, Queen's Medical Centre, Nottingham University Hospitals Trust and University of Nottingham, Nottingham, NG7 2UH, UK; 2 The Breakthrough Breast Cancer Research Centre, Institute of Cancer Research, London, SW3 6JB, UK; 3 Division of Epidemiology and Public Health, School of Community Health Sciences, Queen's Medical Centre, Nottingham University Hospitals Trust and University of Nottingham, Nottingham, NG7 2UH, UK

## Abstract

**Background:**

The amplicon on 8p11.2 is reported to be found in up to 10% of breast carcinomas. It has been demonstrated recently that this amplicon has four separate cores. The second core encompasses important oncogene candidates, including the fibroblast growth factor receptor 1 (*FGFR1*) gene. Recent studies have demonstrated that specific *FGFR1 *amplification correlates with gene expression and that FGFR1 activity is required for the survival of a *FGFR1 *amplified breast cancer cell line.

**Methods:**

*FGFR1 *amplification was analysed in tissue microarrays comprising a cohort of 880 unselected breast tumours by means of chromogenic *in situ *hybridisation using inhouse-generated *FGFR1*-specific probes. Chromogenic *in situ *hybridisation signals were counted in a minimum 30 morphologically unequivocal neoplastic cells. Amplification was defined as >5 signals per nucleus in more than 50% of cancer cells or when large gene copy clusters were seen.

**Results:**

*FGFR1 *amplification was observed in 8.7% of the tumours and was significantly more prevalent in patients >50 years of age and in tumours that lacked HER2 expression. No association was found with other histological parameters. Survival analysis revealed *FGFR1 *amplification as an independent prognostic factor for overall survival in the whole cohort. Subgroup analysis demonstrated that the independent prognostic impact of *FGFR1 *amplification was only seen in patients with oestrogen-receptor-positive tumours, where *FGFR1 *amplification was the strongest independent predictor of poor outcome.

**Conclusion:**

Given that up to 8.7% of all breast cancers harbour *FGFR1 *amplification and that this amplification is an independent predictor of overall survival, further studies analysing the *FGFR1 *as a potential therapeutic target for breast cancer patients are warranted.

## Introduction

Amplification of 8p11.2-p12 is reported to be found in up to 10–15% of all breast cancers [1-5]. For a long time it was believed that the fibroblast growth factor receptor 1 (*FGFR1*) would be the target oncogene of 8p11.2-p12 amplifications [1,2,5,6]. Recent studies, however, have called into question the role of *FGFR1 *as the 'amplicon driver', given that not all cell lines and breast cancers with 8p11.2-p12 amplification overexpressed the *FGFR1 *gene and that FGFR1 protein and mRNA expression was much more pervasive than gene amplification [7-9]. Other oncogene candidates for the amplicon on 8p11.2-p12 have been put forward, including zinc finger protein 703 (*FLJ14299*), SPFH domain family member 2 (*SPFH2*, also known as *C8orf2*), subunit of RNA polymerase III transcription initiation factor (*BRF2*) and RAB11 family interacting protein 1 (*RAB11FIP1*) [7].

Gelsi-Boyer and colleagues [3] have demonstrated more recently that the 8p11.2-p12 amplicon is much more complex than previously anticipated and that it comprises at least four independent cores, which can be amplified independently [3]. While *FLJ14299*, *SPFH2*, proline synthetase cotranscribed homologue (*PROSC*), *BRF2*, and *RAB11FIP1 *were associated with core A1, golgin subfamily a 7 (*GOLGA7*) was correlated with A3, and MYST histone acetyltransferase 3 (*MYST3*) and miR172-resistant version of AP2 (*AP2M3*) were associated with A4. The genes whose expression correlated with amplification of core A2 included LSM1 homologue, U6 small nuclear RNA associated (*LSM1*, also known as cancer-associated Sm-like (*CASM*)), DDHD domain containing 2 (*DDHD2*), phosphatidic acid phosphatase type 2 domain containing 1B (*HTPAP*), Wolf–Hirschhorn syndrome candidate 1-like 1 (*WHSC1L1*), *TM2 *and *FGFR1 *[3]. Interestingly, amplification of core 2, but not the other cores, was associated with shorter metastasis-free survival [3].

*FGFR1 *gene encodes a tyrosine kinase receptor that is part of the fibroblast growth factor and growth factor receptors family [10-12]. FGFR1 expression has been shown to play pivotal roles in mammary development and breast cancer tumourigenesis [12,13]. Activation of FGFR1 in a transgenic mouse model resulted in increased luminal cell proliferation, activation of mitogen-activated protein kinase and Akt, lateral budding and, eventually, alveolar hyperplasia and invasive lesions [11,12]. In addition, we have recently demonstrated that when core 2 of the 8p11.2-p12 is amplified, the *FGFR1 *gene shows increased levels of mRNA and protein expression [14]. Furthermore, we have also determined *in vitro *that FGFR1 signalling is paramount for the survival of a *FGFR1 *amplified breast cancer cell line [14].

The prognostic impact of *FGFR1 *amplification in breast cancer still remains unclear. In previous studies analysing *FGFR1 *amplification by means of Southern blot or fluorescent *in situ *hybridisation, conflicting results were observed: while *FGFR1 *amplification proved to be associated with positivity for oestrogen receptor (ER) in one study [1], Prentice and colleagues found no association between *FGFR1 *amplification and clinicopathological parameters or patients' survival [4]. More recently, using probes for both *RAB11FIP1 *and *FGFR1*, Letessier and colleagues [15] demonstrated that cases with 8p12 amplification have a significantly shorter metastasis-free survival [15].

Chromogenic *in situ *hybridisation (CISH) is a technique that allows for a concurrent analysis of the gene copy number and morphological features of the cells [16-21]. Although results obtained with CISH show an excellent concordance with those obtained with fluorescent *in situ *hybridisation [21], CISH has proven useful for high-throughput copy number assessment, given that it can be easily applied to tissue microarrays and the analysis can be performed with a conventional light microscope [16-21]. No studies analysing *FGFR1 *amplification, as defined by CISH, in a large cohort of breast cancer patients have so far been performed. Using a previously described method [18], inhouse probes specific for *FGFR1 *were generated and we set out to characterise the prevalence of *FGFR1 *amplification in a large community-based cohort of breast cancers and its correlations with traditional clinicopathological features, immunohistochemical markers, and disease-free and overall survival.

## Materials and methods

### Tissue microarrays

The tissue microarrays comprised a cohort of 880 unselected breast tumours from patients presenting between 1986 and 1998 entered into the Nottingham Tenovus Primary Breast Carcinoma Series (447 invasive ductal carcinomas of no special type, 183 tubular mixed carcinomas, 25 medullary carcinomas, 84 lobular carcinomas, 28 tubular carcinomas, eight mucinous carcinomas, six cribriform carcinomas, four papillary carcinomas, 29 mixed no special type and lobular carcinomas, 23 mixed no special type and special-type carcinomas, and six miscellaneous tumours – histological type not available in 37 cases). Patient management was based on tumour characteristics by the Nottingham Prognostic Index (NPI) and hormone receptor status. Patients with an NPI score ≤3.4 received no adjuvant therapy, and those with a NPI score >3.4 received tamoxifen if they were ER-positive (± Zoladex if premenopausal) or received classical cyclophosphamide, methotrexate and 5-fluorouracil if they were ER-negative and fit enough to tolerate chemotherapy [22]. Full details of the characterisation of the tissue microarray and the cohort of patients are summarised in Table 1. Tumours were graded according to a modified Bloom–Richardson scoring system [23] and size was categorised according to the TNM staging criteria [24]. The NPI was calculated as previously described [25].

Survival data including the survival time and the disease-free interval were maintained on a prospective basis. Disease-free survival was defined as the interval (in months) from the date of the primary surgical treatment to the first locoregional (including invasive malignancy and ductal carcinoma *in situ*) or distant recurrence. Overall survival was taken as the time (in months) from the date of the primary surgical treatment to the time of death from breast cancer. The immunohistochemical methods and the results on ER, progesterone receptor, cytokeratin (CK) 7/8, CK 18, CK 19, CK 5/6, CK 14, HER2 and epidermal growth factor receptor have been previously described [26,27].

This study was approved by the Nottingham Research Ethics Committee 2 under the title 'Development of a molecular genetic classification of breast cancer'.

### Chromogenic *in situ *hybridisation

CISH for *FGFR1 *gene amplification was performed on 2-μm-thick tissue microarray sections mounted on polylysine-coated slides, using an inhouse-generated probe as previously described [18]. This probe comprises three bacterial artificial chromosome contigs (RP11-350N15, RP11-148D21 and RP11-359P11), which map to the region 38.3–38.6 Mb on chromosome 8p12-p11.23 and encompasses the *FGFR1 *and part of *WHSC1L1*. Heat pretreatment of deparaffinised sections consisted of incubation for 15 minutes at 98°C in CISH pretreatment buffer (SPOT-light tissue pretreatment kit; Zymed (South San Francisco, CA, USA) and digestion with pepsin for 5.5 minutes at room temperature according to the manufacturer's instructions. Slides were hybridised and developed as previously described. An appropriate *FGFR1 *gene-amplified breast carcinoma control was included in the slide run.

CISH experiments were analysed by two of the authors (SEE and ARG) on a multiheaded microscope. Only unequivocal signals were counted. Signals were evaluated at 400 × magnification and 630 × magnification, and 30 morphologically unequivocal neoplastic cells in each core were assessed for the presence of the *FGFR1 *gene signals. Amplification was defined as those cases where >50% of the neoplastic cells harboured either >5 copies of the gene or large gene clusters. CISH analysis was performed with observers blinded to clinicopathological parameters, patients' survival and results of the immunohistochemical analysis.

### Statistical analysis

Statistical analysis was performed using SPSS 13.0 statistical software (SPSS INC., Chicago, IL, USA). Median follow-up was defined as the median follow-up for those patients still alive and disease free at the latest hospital visit. Cutoff values for the different biomarkers included in this study were chosen before statistical analysis. Standard cutoff values were used for established prognostic factors and were the same as for previously published patient series [27]. All factors were used as dichotomous covariates in the statistical analysis with the exception of grade and the NPI, which were categorised into three groups.

The associations between the *FGFR1 *amplification and clinicopathological parameters were evaluated by the chi-square test. Confidence intervals of 95% were adopted. A two-sided *P *value <0.05 was considered statistically significant. Survival curves were calculated by the Kaplan–Meier method. Differences in survival on the basis of *FGFR1 *amplification were estimated using the log-rank test. Multivariate Cox regression analysis was used to evaluate any independent prognostic effect of the variable on disease-free survival and the overall survival, which was adjusted by such well-known prognostic factors as tumour grade, lymph node stage, tumour size, and ER status.

## Results

**Figure 1:**
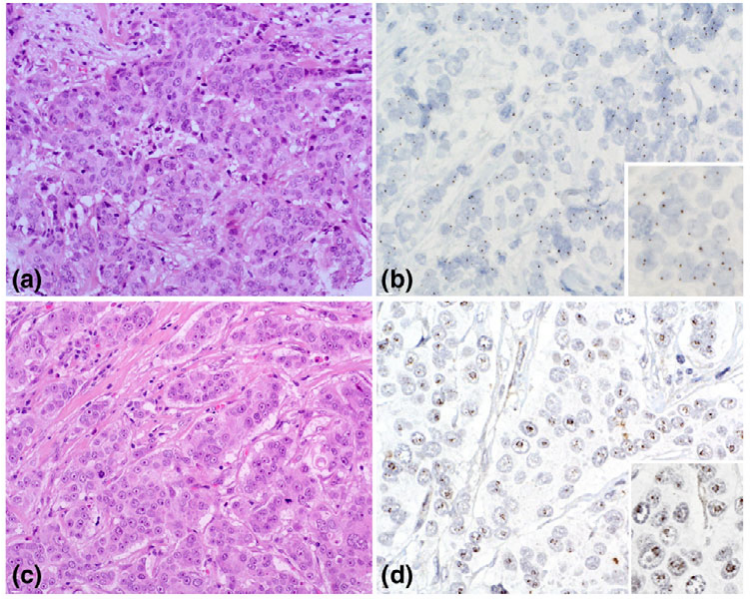
*FGFR1 *gene amplification in breast cancer. **(a) **Grade 3 invasive ductal carcinoma (haematoxylin and eosin; original magnification × 200) with **(b) **one or two copies of FGFR1 (original magnification × 400; inset: × 630). **(c) **Grade 3 invasive ductal carcinoma (haematoxylin and eosin; original magnification × 200) harbouring **(d) ***FGFR1 *gene amplification (original magnification × 400; inset: × 630).

After excluding the uninformative tissue microarray cores, results on *FGFR1 *amplification were available for 496 tumours. Forty-three tumours (8.7%) showed either >5 signals or large gene clusters in >50% of neoplastic cells (Figure 1).

Complete clinical follow-up information was available for 478 patients for whom *FGFR1 *CISH results were optimal. The median follow-up period was 58 months (range 1–192 months). During this period, a total of 73 (14.7%) patients died from breast cancer. Of all cases, 116 (24.3%) cases were grade 1, 141 (29.5%) cases were grade 2, and 221 (46.2%) were grade 3. From the available data, 153 (32.2%) of the patients had lymph-node-positive disease, 130 (27.8%) had positive vascular invasion, and 311 (65.1%) had tumour size ≥1.5 cm. Recurrence occurred in 147 cases (30.8%), and distant metastases developed in 84 cases (17.6%). A total of 149/496 (30%) patients received tamoxifen, 5/496 (1%) received tamoxifen and Zoladex, and 309/496 (62.3%) received no endocrine treatment. There was no significant difference between the disease-free interval (*P *= 0.761) or overall survival (*P *= 0.225) between those patients that received hormone therapy compared with those patients that did not. A total of 48/496 (9.7%) patients received chemotherapy.

Table 2 summarises the associations between *FGFR1 *gene amplification and key prognostic and outcome parameters. In brief, patients with positive *FGFR1 *amplification were significantly more likely to be older than 50 years of age (*P *< 0.05) and to develop distant metastasis (*P *< 0.05). *FGFR1 *amplification showed an inverse correlation with HER2 overexpression. A trend for lack of progesterone receptor expression and negativity for basal markers, as defined by Abd El-Rehim and colleagues [27], was also observed. No associations were found between *FGFR1 *amplification and the grade, lymph-node stage, NPI, expression of ER, low-molecular-weight cytokeratins (CK 7/8, CK 8 and CK 19) or high-molecular-weight cytokeratins (CK 5/6 and CK 14) or basal-like phenotype as defined by Nielsen and colleagues [28] (that is, ER-negative and HER2-negative, CK 5/6 and/or epidermal growth factor receptor-positive).

**Figure 2:**
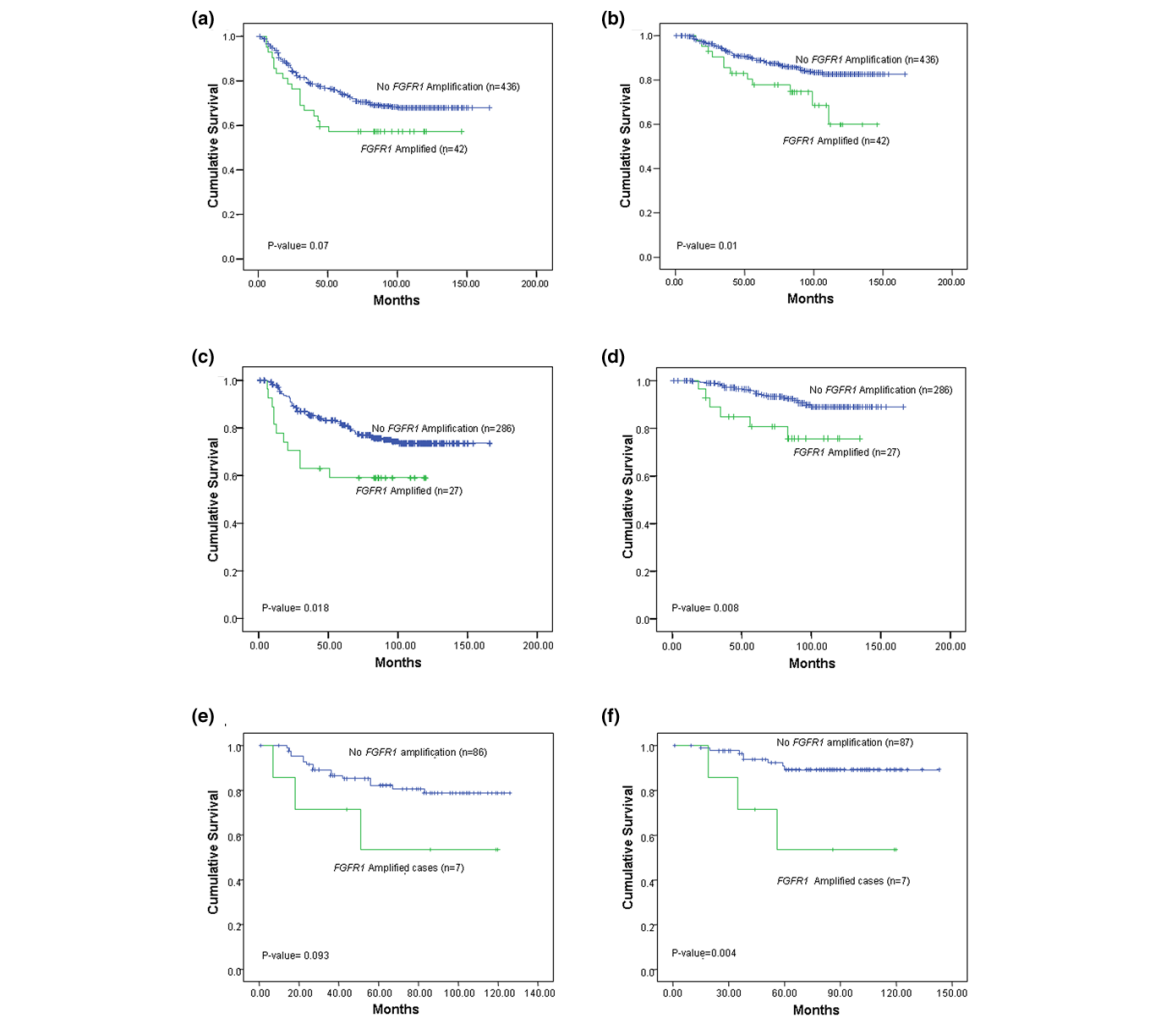
Univariate analysis of the prognostic impact of *FGFR1 *gene amplification overexpression on disease-free survival and overall survival. **(a) **Whole cohort, disease-free survival. **(b) **Whole cohort, overall survival. **(c) **Oestrogen-receptor-positive cases, disease-free survival. **(d) **Oestrogen-receptor-positive cases, overall survival. **(e) **Oestrogen-receptor-positive patients that received endocrine therapy, disease-free survival. **(f) **Oestrogen-receptor-positive patients that received endocrine therapy, overall survival.

Kaplan–Meier survival analysis revealed an association between *FGFR1 *amplification and a shorter overall survival (*P *= 0.01, log-rank test) (Figure 2a). A trend for a shorter disease-free survival and *FGFR1 *amplification was found (*P *< 0.07, log-rank test) (Figure 2b). On multivariate Cox hazard analysis adjusted for tumour grade, size and lymph node status, for ER status, and for *FGFR1 *amplification, it was found that the *FGFR1 *amplification was a significant predictor of poor overall survival independent of the other known prognostic parameters (*P *< 0.04) (Table 3).

Subgroup analysis revealed that *FGFR1 *amplification was an independent prognostic factor for disease-free survival and overall survival only in ER-positive tumours (Figure 2c,d and Table 4). The association between *FGFR1 *amplification and poor outcome was maintained in the group of patients that received endocrine therapy (Figure 2e,f). *FGFR1 *amplification in ER-positive disease was the strongest independent risk factor for poor disease-free survival and overall survival, with a greater hazard ratio than high histological grade. Patients with *FGFR1 *amplification in the ER-positive group were significantly more likely to develop distant metastases and were associated with a lack of progesterone receptor expression (*P *< 0.05) (Table 5). In the cohort of ER-positive tumours, no further associations between *FGFR1 *amplification and other clinicopathological parameters were found. No associations were seen between *FGFR1 *amplification and survival of patients with ER-negative breast cancers.

## Discussion

In recent years it has been demonstrated that CISH is a useful technique to determine gene copy numbers and gene amplification on formalin-fixed, paraffin-embedded tissue sections [16-21]. Unlike fluorescent *in situ *hybridisation, CISH allows a direct comparison between morphological features of neoplastic cells and the presence of gene amplification [16-21]. Furthermore, CISH analysis is relatively quick; in the present study, the whole analysis of *FGFR1 *amplification in a cohort of 880 patients took 2 weeks and, although only one tissue microarray core per tumour was analysed, 56% cases rendered optimal results.

We demonstrate in the present study that *FGFR1 *amplification is found in 8.7% of breast cancers, which is in agreement with previous studies [1-4,29]. Unlike previous studies where *FGFR1 *amplification was determined by Southern blot analysis [1], our results and those obtained with other *in situ *methods [3,4,15] did not show any correlation between *FGFR1 *amplification and low histological grade or positivity for ER. On the other hand, our results demonstrate that *FGFR1 *amplification is an independent predictor of poor outcome, especially for patients with ER-positive breast cancers. Interestingly, the impact of *FGFR1 *amplification was stronger on overall survival than disease-free survival (that is, higher hazard ratios on multivariate analysis). This may stem from the fact that locoregional recurrences were included as events for disease-free survival analysis, and that overall survival considered only breast-cancer-related deaths as events. Alternatively, this may reflect the association between *FGFR1 *amplification and the development of distant metastasis (*P *= 0.05) or a shorter survival after the first distant recurrence event.

Our group [14] and others [3] have demonstrated that when *FGFR1 *is specifically amplified (that is, amplification of core A2 of the 8p11.2-p12 amplicon) it is also overexpressed, and that *FGFR1 *signalling is important for the survival of a cell line that harbours *FGFR1 *amplification and high-level gene expression [14,30]. Taken together, these results suggest that, in a significant proportion of cases with core A2 amplification, *FGFR1 *may be the actual amplicon driver [6]. We could not correlate *FGFR1 *amplification with expression in this study, as it was not possible to optimise antibodies for FGFR1 immunohistochemical analysis on tissue microarrays due to the highly fixation-dependent nature of the commercially available antibodies (data not shown).

The *FGFR1 *gene encodes a tyrosine kinase receptor that has been shown to play an important role in mammary gland development [12,13,31]. Previous studies have shown *in vitro *and *in vivo *that *FGFR1 *overexpression has oncogenic properties [10,12-14,31]. Furthermore, *FGFR1 *has been implicated in the tumourigenesis of haematological malignancies, where it is frequently involved in balanced chromosomal translocations, including cases of chronic myeloid leukaemia (*BCR*-*FGFR1 *fusion) and the 8p11 myeloproliferative syndrome/stem cell leukaemia–lymphoma syndrome, which is characterised by myeloid hyperplasia and non-Hodgkin's lymphoma with chromosomal translocations fusing several genes, the most common being a fusion between *ZNF198 *and *FGFR1 *[32]. In preclinical models, the PKC412 tyrosine kinase inhibitor has been shown to successfully inhibit the growth of proliferation of *ZNF198*-*FGFR1*-transformed Ba/F3 cells and to prolong the survival of animals with a ZNF198-FGFR1-induced stem cell leukaemia–lymphoma syndrome [33]. Targeting FGFR1 signalling with RNA interference or with the SU5402 FGFR1 tyrosine kinase inhibitor has been shown to decrease cell survival in a breast cancer cell line with *FGFR1 *amplification [14].

## Conclusion

Taken together, our results demonstrate that *FGFR1 *amplification is found in 8.7% of breast cancers and is an independent predictor of outcome. Although large studies correlating *FGFR1 *amplification with mRNA and protein expression are still needed, the functional data demonstrating that FGFR1 signalling is required for the survival of breast cancer cells harbouring *FGFR1 *amplification [14], the relatively high prevalence of *FGFR1 *amplification in breast cancer and the independent prognostic information provided by *FGFR1 *amplification status support the idea that this gene may be a useful therapeutic target for a subgroup of breast cancer patients with *FGFR1 *gene amplification [14].

## Abbreviations

CISH = chromogenic *in situ *hybridisation; CK = cytokeratin; ER = oestrogen receptor; FGFR1 = fibroblast growth factor receptor 1; NPI = Nottingham Prognostic Index.

## Competing interests

The authors declare that they have no competing interests.

## Authors' contributions

SEE and ARG analysed the CISH experiments, performed in part the statistical analysis and helped to draft the manuscript. MBKL performed the laboratory work for *FGFR1 *inhouse probe generation and hybridisation to tissue microarrays. NCT participated in the study design, the interpretation of the results and statistical analysis. MJG supervised and reviewed the statistical analysis. DP participated in the interpretation of the results. IOE participated in the study design, data analysis and helped to draft the manuscript. JSR-F conceived the study, designed the probes for *FGFR1*, supervised the statistical analysis and drafted the manuscript. All authors read and approved the final manuscript.

## Figures and Tables

**Table 1:** Frequencies and percentages of tumour grades, size, lymph node stage, distant metastasis and patient age in the tissue microarrays

	Frequency	Percentage
Grade		
1	191	22.7
2	273	32.4
3	378	44.9
Lymph node stage		
0	561	66.7
N1	203	24.1
N2	77	9.2
Tumour size		
≤1.5 cm	301	35.7
>1.5 cm	543	64.3
Distance metastasis		
M0	717	85.0
M1	127	15.0
Age		
≤50 years	299	35.4
>50 years	545	64.6

**Table 2:** Correlation between *FGFR1 *gene amplification and the clinicopathological variables

Parameter	Number of samples	Positive FGFR1 amplification number (%)	*P *value
Age	478		0.027
<50 years	152	7 (4.6)	
≥50 years	346	35 (10.1)	
Grade	478		Not significant
1	116	10 (8.6)	
2	141	12 (8.5)	
3	221	20 (9.0)	
Size	478		Not significant
≥1.5 cm	311	30 (9.6)	
>1.5 cm	167	12 (7.2)	
Lymph node stage	475		Not significant
N0	322	28 (8.7)	
N1	110	9 (8.2)	
N2	43	4 (9.3)	
Nottingham Prognostic Index	475		Not significant
Good	175	14 (8.0)	
Moderate	239	21 (8.8)	
Poor	61	6 (9.8)	
Distant metastasis	478		0.050
No	394	30 (7.6)	
Definite	84	12 (14.3)	
Vascular invasion	467		Not significant
No	337	27 (8.0)	
Yes	130	14 (10.8)	
Oestrogen receptor	453		Not significant
Negative	140	10 (7.1)	
Positive	313	27 (8.6)	
Progesterone receptor	449		0.084
Negative	206	22 (10.7)	
Positive	243	15 (6.2)	
HER2	445		0.043
Negative	259	28 (10.8)	
Positive	186	10 (5.4)	
Androgen receptor	415		Not significant
Negative	188	18 (9.6)	
Positive	227	13 (5.7)	
Cytokeratin 7/8	467		Not significant
Negative	167	17 (10.2)	
Positive	300	24 (8.0)	
Cytokeratin 18	396		Not significant
Negative	116	7 (6.0)	
Positive	280	23 (8.2)	
Cytokeratin 19	464		Not significant
Negative	95	10 (10.5)	
Positive	369	30 (8.1)	
Cytokeratin 5/6	465		Not significant
Negative	350	34 (9.7)	
Positive	115	6 (5.2)	
Cytokeratin 14	458		Not significant
Negative	357	33 (9.2)	
Positive	101	6 (5.9)	
Epidermal growth factor receptor	351		Not significant
Negative	292	26 (8.9)	
Positive	59	3 (5.1)	
Basal markers [27]	455		Not significant
Negative	311	31 (10.0)	
Positive	144	8 (5.6)	
Nielsen groups [28]	396		Not significant
HER2	39	3 (7.7)	
Basal-like	55	3 (5.5)	
Luminal	302	26 (8.6)	

**Table 3:** Cox proportional hazards analysis for predictors of overall survival: effects of tumour grade, size, lymph node stage, oestrogen receptor status and *FGFR1 *amplification

Variable	Hazard ratio	95% confidence interval	*P *value
Grade^a^	2.13	1.32–3.44	0.002
Tumour size ≥1.5 cm^b^	2.17	1.06–4.44	0.034
Positive lymph node stage	3.52	2.17–5.72	<0.001
Positive oestrogen receptor status	0.44	0.26–0.74	0.002
FGFR1 amplification	2.00	1.02–3.92	0.043

^a^Fitted as a linear term; that is, an increase in risk for change in grade of one unit.

^b^Compared with tumour size <1.5 cm.

**Table 4:** Cox proportional hazards analysis for predictors of disease-free and overall survival: effects of tumour grade, size, lymph node stage, oestrogen receptor (ER) status and FGFR1 status in ER-positive and ER-negative tumours

Variable	ER-positive tumours			ER-negative tumours		
	
	Hazard ratio	95% confidence interval	*P *value	Hazard ratio	95% confidence interval	*P *value
Disease-free interval						
Grade^a^	1.387	1.027–1.874	0.033	1.001	0.637–1.574	0.996
Tumour size ≥1.5 cm^b^	1.551	0.926–2.595	0.095	2.348	1.092–5.052	0.029
Positive lymph node stage	1.407	0.879–2.252	0.155	2.597	1.563–4.315	0.000
FGFR1 amplification	2.250	1.184–4.274	0.013	1.104	0.440–2.774	0.833
Overall survival						
Grade^a^	2.600	1.471–4.594	0.001	1.673	0.731–3.829	0.223
Tumour size ≥1.5 cm^b^	2.124	0.792–5.697	0.134	2.594	0.911–7.387	0.074
Positive lymph node stage	1.973	0.953–4.083	0.067	5.124	2.646–9.924	0.000
FGFR1 amplification	3.389	1.374–8.358	0.008	1.278	0.450–3.624	0.645

^a^Fitted as a linear term; that is, an increase in risk for change in grade of one unit.

^b^Compared with tumour size <1.5 cm.

**Table 5:** Correlation between *FGFR1 *gene amplification and the clinicopathological variables in the oestrogen-receptor-positive group

Parameter	Number of samples	Positive FGFR1 amplification number (%)	*P *value
Age	313		Not significant
<50 years	95	6 (6.3)	
≥50 years	218	21 (9.6)	
Grade	313		Not significant
1	99	8 (8.1)	
2	120	10 (8.3)	
3	94	9 (9.6)	
Size	313		Not significant
≥1.5 cm	188	19 (10.1)	
<1.5 cm	125	8 (6.4)	
Lymph node stage	311		Not significant
N0	214	19 (8.9)	
N1	74	6 (8.2)	
N2	23	2 (9.3)	
Nottingham Prognostic Index	311		Not significant
Good	148	11 (7.4)	
Moderate	133	14 (10.5)	
Poor	30	2 (6.7)	
Distant metastasis	313		0.032
No	273	20 (7.3)	
Definite	40	7 (17.5)	
Vascular invasion	303		Not significant
No	224	18 (8)	
Yes	79	8 (10.1)	
Progesterone receptor	305		0.05
Negative	80	11 (13.8)	
Positive	225	15 (6.7)	
HER2	299		0.063
Negative	179	20 (11.2)	
Positive	120	6 (5.0)	
Androgen receptor	283		0.08
Negative	90	11 (12.2)	
Positive	193	12 (6.2)	
Cytokeratin 7/8	310		Not significant
Negative	73	8 (11)	
Positive	237	19 (8.0)	
Cytokeratin 18	269		Not significant
Negative	46	1 (2.2)	
Positive	223	21 (9.4)	
Cytokeratin 19	308		Not significant
Negative	33	4 (12.1)	
Positive	275	22 (8.0)	
Cytokeratin 5/6	306		Not significant
Negative	261	24 (9.2)	
Positive	45	2 (4.4)	
Cytokeratin 14	299		Not significant
Negative	252	23 (9.1)	
Positive	47	2 (4.3)	
Epidermal growth factor receptor	242		Not significant
Negative	209	19 (9.1)	
Positive	33	2 (6.1)	
Basal markers [27]	297		Not significant
Negative	234	22 (9.4)	
Positive	63	3 (4.8)	
